# Mitochondria-dependent signalling pathway are involved in the early process of radiation-induced bystander effects

**DOI:** 10.1038/sj.bjc.6604358

**Published:** 2008-05-13

**Authors:** S Chen, Y Zhao, W Han, G Zhao, L Zhu, J Wang, L Bao, E Jiang, A Xu, T K Hei, Z Yu, L Wu

**Affiliations:** 1 Key Laboratory of Ion Beam Bioengineering, Institute of Plasma Physics, Chinese Academy of Sciences, Hefei 230031, People's Republic of China; 2 Center for Radiological Research, College of Physicians and Surgeons, Columbia University, New York, NY 10032, USA

**Keywords:** radiation-induced bystander effects, signalling pathway, mitochondrion, nitric oxide synthase

## Abstract

Bystander effects induced by cytoplasmic irradiation have been reported recently. However, the mechanism(s) underlying, such as the functional role of mitochondria, is not clear. In the present study, we used either mtDNA-depleted (*ρ*^0^) A_L_ or normal (*ρ*^+^) A_L_ cells as irradiated donor cells and normal human skin fibroblasts as receptor cells in a series of medium transfer experiments to investigate the mitochondria-related signal process. Our results indicated that mtDNA-depleted cells or normal A_L_ cells treated with mitochondrial respiratory chain function inhibitors had an attenuated *γ*-H2AX induction, which indicates that mitochondria play a functional role in bystander effects. Moreover, it was found that treatment of normal A_L_ donor cells with specific inhibitors of NOS, or inhibitor of mitochondrial calcium uptake (ruthenium red) significantly decreased *γ*-H2AX induction and that radiation could stimulate cellular NO and O_2_^•−^ production in irradiated *ρ*^+^ A_L_ cells, but not in *ρ*^0^ A_L_ cells. These observations, together with the findings that ruthenium red treatment significantly reduced the NO and O_2_^•−^ levels in irradiated *ρ*^+^ A_L_ cells, suggest that radiation-induced NO derived from mitochondria might be an intracellular bystander factor and calcium-dependent mitochondrial NOS might play an essential role in the process.

Although radiation-induced bystander effects (RIBEs) have generated a lot of interest in the field of radiation biology, the mechanism(s) underlying the bystander phenomenon are not quite clear. Emerging evidence indicates that soluble transmissible factor(s) and gap junction-mediated cell–cell communications are critical in mediating the bystander effects (Mothersill and Seymour, 1998; Azzam *et al*, 2001). Moreover, there is evidence that cytoplasmic irradiation may also be capable of inducing the bystander signalling molecules. Earlier report by Wu *et al* (1999) indicated that irradiating only cytoplasm resulted in CD59 locus mutations in the human–hamster hybrid (A_L_) cells. Recently, Shao *et al* (2004) reported that when a single cell within the glioma population was traversed through its cytoplasm with one helium ion, bystander response was induced in the neighbouring, nonirradiated glioma or fibroblasts. In our previous studies, we demonstrated that the induction of excessive *γ*-H2AX could be detected in bystander cells within minutes after irradiation (Hu *et al*, 2005; Han *et al*, 2007b), and NO produced by constitutive nitric oxide synthase (cNOS) was identified as the possible intercellular signalling molecule to initiate and activate bystander signalling pathway in the early process (Han *et al*, 2007a). However, the exact locations where the signalling molecule(s), such as reactive oxygen/nitrogen species (RNS/ROS), are generated in the cytoplasm and what is the role of mitochondria in the early processes of RIBE remain unknown.

Mitochondria occupy a unique position among cellular organelles and play an important role in the generation of free radicals and in the regulation of apoptosis (Balaban *et al*, 2005). Ionising radiation has been found to induce mitochondrial damage by increase of ROS production, depolarisation of mitochondrial membrane potential, and release of cytochrome *c* in directly irradiated cells (Leach *et al*, 2001). There is also evidence that irradiated cell conditioned media (ICCM) can cause changes of mitochondrial distribution, loss of mitochondrial membrane potential, increases in ROSs, and increase in apoptosis among the medium receptor cells, which can be blocked by treatments with antioxidants (Lyng *et al*, 2000; Maguire *et al*, 2005). Furthermore, Limoli *et al* (2003) have reported that a state of chronic oxidative stress derived in part from dysfunctional mitochondria may be linked to many of the abnormal phenotypes associated with genomic instability in the progeny of irradiated cells.

In the present studies, we investigated the role of mitochondria in the formation and transduction of signals during the early stage of the bystander process. To address these goals, a medium transfer approach was adopted, and normal A_L_ cells (*ρ*^+^) and mitochondrial DNA (mtDNA)-depleted A_L_ cells (*ρ*^0^) were used as medium donor cells, whereas AG1522 cells were used as the medium receptor cells. With the detection of *γ*-H2AX foci induction in receptor cells and the determination of the origin of NO and O_2_^•−^ in irradiated cells, our results suggested that mitochondria-derived NO and O_2_^•−^ played an important role in the initiation and activation of the early process (⩽30 min) of RIBE.

## MATERIALS AND METHODS

### Cell culture

Two hamster cell lines: normal A_L_ cells (*ρ*^+^) and mtDNA-depleted A_L_ cells (*ρ*^0^) together with normal human fibroblasts in passage 11–14 (AG1522) were used in the present studies. A_L_ cells (*ρ*^0^) were generated in the laboratory of one of the co-authors (TK Hei) by treating normal A_L_ cells with the chemotherapeutic drug ditercalinium over a period of 3–4 months to deplete the mtDNA by >95% (Liu *et al*, 2005). All cells were cultured and maintained at 37°C in a humidified 95% air/5% CO_2_ incubator. For media transfer study, *ρ*^+^ A_L_ cells and *ρ*^0^ A_L_ cells were used as medium donor cells after irradiation, and AG1522 cells were used as medium receptor cells.

### *α*-Particle irradiation and medium transfer experiments

The average energy and LET of *α*-particles derived from the ^241^Am irradiation source of the radiation facility, measured at the cell layer, was 3.5 MeV and 128 keV *μ*m^−1^ and the particles were delivered at a dose rate of 1.0 cGy s^−1^. For medium transfer experiment, *ρ*^+^ A_L_ cells and *ρ*^0^ A_L_ cells under confluent conditions in a 35-mm stainless steel dish with a 3.5-*μ*m-thick replaceable mylar bottom were placed on the rotating sample bracket and irradiated with a 1-cGy dose of *α*-particles. After irradiation, the cultures were put back into the incubator for 10 min. Then, the medium from the irradiated population was collected, filtered through a 0.8-*μ*m syringe filter, and transferred immediately into a rectangular dish (10 × 6 mm^2^) full of nonirradiated, confluent AG1522 cells (>92% cells in G1 phase). Thereafter, these receptor cells were incubated for 30 min with ICCM and then fixed with 2% paraformaldehyde solution for immunostaining. Medium from sham-irradiated dish was transferred to the receptor cells as controls.

### Immunostaining for *γ*-H2AX

For the immunostaining, the fixed cells were permeabilised in TNBS solution (PBS supplemented with 0.1% Triton X-100 and 1% FBS), followed by exposing cells to anti-*γ*-H2AX primary antibody (Upstate Biotechnology, Lake Placid, New York, USA) for 1 h. Then, the cells were incubated with fluorescein isothiocyanate-conjugated goat anti-mouse secondary antibody (Haoyang Biological Manufacture Company, Tianjin, China) for another 1 h. After washing with TNBS for 3 × 5 min, cells were counterstained with 5 mg ml^−1^ Hoechst 33342 (Molecular Probes, Eugene, Oregon, USA).

Immunofluorescent images were captured by confocal laser scanning microscope (TCS SP2; Leica, Wetzlar, Germany). For quantitative analysis, the cells with at least one *γ*-H2AX focus were regarded as the positive cells and the fraction of positive cells was calculated (cells with *γ*-H2AX foci/total cells) (Limoli *et al*, 2002; Hu *et al*, 2005). At least 700 cells in each sample were counted, and the fraction of positive cells of irradiated groups was normalised to their nonirradiated controls. Statistical analyses were performed on the means of the data obtained from at least three independent experiments.

### *γ*-H2AX induction in receptor cells after pretreatment with inhibitors of mitochondrial respiratory chain function

Before irradiation, the medium in the *ρ*^+^ A_L_ donor cells was replaced with fresh medium containing either 1 *μ*M rotenone (Sigma, Steinheim, Germany) for 30 min, 5 *μ*g ml^−1^ antimycin A (Sigma, Steinheim, Germany) for 2 h, or 10 *μ*M oligomycin (Sigma, Steinheim, Germany) for 1 h to inhibit respiratory chain complexes I, III, or V, respectively. After washing twice with D-Hanks buffer, and being replenished with fresh media, cells were irradiated with 1 cGy *α*-particles, and the media were, subsequently, transferred to receptor cells 10 min later as described previously.

### *γ*-H2AX induction in receptor cells after pretreatment with inhibitors of nitric oxide synthase

The medium in the *ρ*^+^ A_L_ donor cells was replaced with fresh media containing 1 mM N^G^-methyl-L-arginine (L-NMMA; Molecular Probes, Eugene, Oregon, USA) for 1 h, 1 mM N^G^-methyl-D-arginine (D-NMMA; Molecular Probes) for 1 h, or 1 *μ*M N^*ω*^-nitro-L-arginine (L-NNA; Sigma, Steinheim, Germany) for 30 min, respectively. N^G^-methyl-L-arginine, is a specific inhibitor of NOS, D-NMMA is the nonreactive D-enantiomer of L-NMMA, and L-NNA is an irreversible inhibitor of cNOS. All inhibitor-treated cells were washed with D-Hanks buffer before irradiation. After irradiation, the donor cells culture was incubated for 10 min, and, then, the medium was immediately collected and transferred to the receptor cells as described above.

### *γ*-H2AX induction in receptor cells after pretreatment with inhibitors of calmodulin and mitochondrial calcium uptake

Confluent *ρ*^+^ A_L_ donor cells were preincubated with 10 *μ*M calmidazolium chloride (Calbiochem, Darmstadt, Germany) for 10 min or 10 *μ*M ruthenium red (RR; Fluka, Steinheim, Germany) for 2 min (Dedkova *et al*, 2004). Calmidazolium chloride is an inhibitor of calmodulin binding with NOS, and RR is an inhibitor of mitochondrial calcium uptake. After irradiation, the media containing inhibitors were removed at once, and cells were incubated in fresh media for 10 min. Finally, the media were transferred into the receptor cells as described above.

### NO and O_2_^•−^ measurement in donor cells

The 4-amino-5-methylamino-2′,7′-difluorofluorescein diacetate (DAF-FM diacetate; Molecular Probes, Eugene, Oregon, USA) and dihydroethidine (Molecular Probes, Eugene, Oregon, USA) were employed to quantify the level of NO and O_2_^•−^, respectively, as described (Narayanan *et al*, 1997; Wan *et al*, 2005; Han *et al*, 2007a). At confluency, cultures (*ρ*^+^ and *ρ*^0^) inoculated on the mylar film in 14-mm stainless steel dishes were stained with 5 *μ*M DAF-FM diacetate in Tyrode's solution or 10 *μ*M dihydroethidine in D-Hanks for 30 min in incubator. The cultures were then irradiated with 1 cGy *α*-particles followed by a 10-min incubation at 37°C. The fluorescence intensity was measured with a fluorescent reader (excitation/emission: 495/515 nm for NO, 488/610 nm for O_2_^•−^), and the fluorescence intensity in 1 cGy-irradiated cultures was normalised to the sham-irradiated cultures. Statistical analysis was performed on the means of the data pooled from at least three independent experiments.

### Effect of mitochondrial calcium uptake on the O_2_^•−^ and NO products

To investigate the effect of mitochondrial calcium uptake on the NO and O_2_^•−^ products in irradiated donor cells, cells preloaded with either DAF-FM diacetate or dihydroethidine were treated with RR for 2 min. The cultures were then irradiated, washed with buffer at once, and fluorescence intensities detected by fluorescence reader as described above.

### Statistical analysis

Data were presented as mean and standard derivations. Comparisons of the *γ*-H2AX induction and fluorescent intensity between treated groups and controls were made by Student's *t*-test. A *P*-value of 0.05 or less between groups was considered to be significant.

## RESULTS

### mtDNA-depletion and inhibition of respiratory chain function attenuate the bystander *γ*-H2AX induction

**Figure 1:**
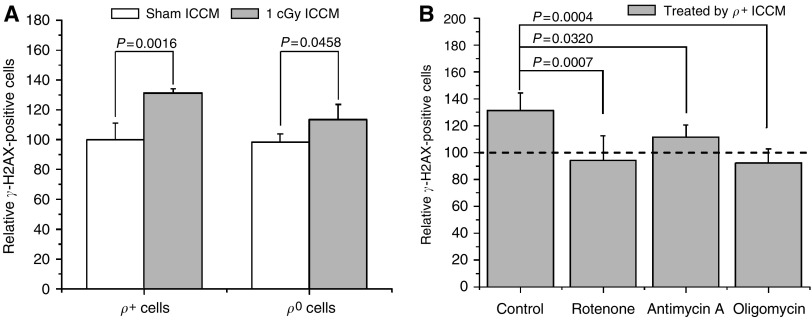
Effects of depleted mtDNA and inhibition of respiratory chain function on the bystander *γ*-H2AX induced by ICCM. The *γ*-H2AX induction ability of *ρ*^+^ ICCM was significantly higher than that of *ρ*^0^ ICCM (1.31±0.03 *vs* 1.13±0.10, *P*<0.01, **A**) and the response was suppressed significantly by inhibitors of respiratory chain (*P*<0.05, **B**). Data are pooled from at least three independent experiments and the results represent mean±s.d.

The background *γ*-H2AX induction in AG1522 cells by ICCM from either *ρ*^+^ or *ρ*^0^ A_L_ cells (*ρ*^+^/*ρ*^0^ ICCM) without irradiation was quite similar (17.1±1.6% for *ρ*^+^ cells and 16.3±1.3% for *ρ*^0^ cells). After irradiation, as shown in Figure 1A, the *γ*-H2AX induction of *ρ*^+^/*ρ*^0^ ICCM were significantly higher than their nonirradiated controls (increased by 31%, *P*<0.01 for *ρ*^+^ cells; increased by 13%, *P*<0.05 for *ρ*^0^ cells). However, the *γ*-H2AX induction of *ρ*^+^ ICCM was significantly higher than that of *ρ*^0^ ICCM (*P*<0.05). These results clearly show that *ρ*^0^ cells have a lower *γ*-H2AX induction ability than wild-type cells and suggest that mitochondria may play a functional role in the early processes of RIBE.

To further investigate if the dysfunction of mitochondrial respiratory chain was related to the attenuated *γ*-H2AX induction of *ρ*^0^ ICCM, *ρ*^+^ A_L_ cells were treated with specific inhibitors of respiratory chain complex before irradiation. As shown in Figure 1B, Rotenone, antimycin A, and oligomycin suppressed the *γ*-H2AX induction in the receptor cells (*P*<0.05). These results indicate that respiratory chain complexes I, III, and V contribute to the generation of early signalling molecules in radiation-induced bystander processes.

### Inhibitors of NOS decrease the bystander *γ*-H2AX induction and radiation stimulates cellular NO production in irradiated *ρ*^+^ A_L_ cells, but not in *ρ*^0^ A_L_ cells

**Figure 2:**
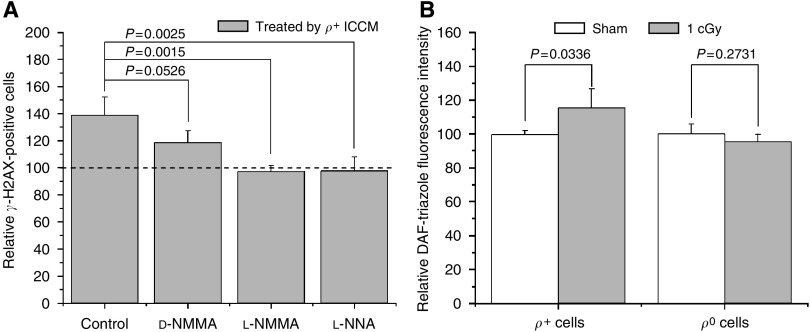
Effect of the NO products on the bystander *γ*-H2AX induction by *ρ*^+^ ICCM. NOS inhibitors, L-NMMA, or L-NNA significantly decreased *ρ*^+^ ICCM-induced bystander*γ*-H2AX (**A**), and fluorescence intensities of DAF-triazole was significantly increased in 1 cGy-irradiated *ρ*^+^ A_L_ cells, but not in 1 cGy-irradiated *ρ*^0^ A_L_ cells (**B**). Data are pooled from at least three independent experiments and the results represent mean±s.d.

To confirm whether the activity of NOS in irradiated *ρ*^+^ A_L_ donor cells is critical in bystander signalling, the donor cultures were treated with L-NMMA, D-NMMA, or L-NNA before irradiation. As shown in Figure 2A, L-NMMA significantly quenched the *γ*-H2AX induction of *ρ*^+^ ICCM in receptor cells (*P*<0.01), whereas the nonreactive D-enantiomer, D-NMMA, employed as negative control, showed no significant effect on *γ*-H2AX induction (*P*>0.05). Similarly, L-NNA significantly inhibited the *γ*-H2AX induction of *ρ*^+^ ICCM in receptor cells (*P*<0.01).

By further measuring the level of NO in *ρ*^+^ or *ρ*^0^ A_L_ cells after irradiation with the fluorescent probes, DAF-DA diacetate, Figure 2B showed that the fluorescence intensities of DAF-triazole, the fluorescent byproduct of DAF-FM, were significantly increased in 1 cGy-irradiated *ρ*^+^ A_L_ cells comparing with the sham-irradiated control (*P*<0.05) and the increased fluorescence intensities of DAF-triazole were caused by NO (Supplementary Figure 1). In contrast, *ρ*^0^ A_L_ cells showed no significant induction relative to controls (*P*>0.05; Figure 2B). These results further confirm that activity of cNOS is causally linked to the early processes of RIBE and that radiation-induced NO products are derived from mitochondria.

### Inhibitors of calmodulin/mitochondrial calcium uptake decrease the bystander *γ*-H2AX induction and radiation-induced NO production

**Figure 3:**
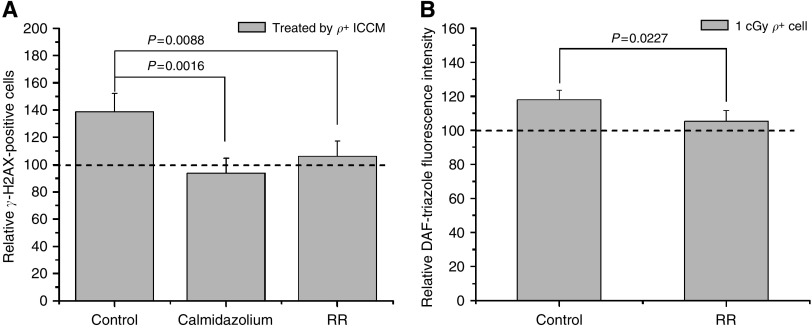
Effect of the calmodulin/Ca^2+^ influx inhibition on the NO products and bystander *γ*-H2AX induction by *ρ*^+^ ICCM. RR or calmidazolium significantly reduced the *γ*-H2AX induction of *ρ*^+^ ICCM (*P*<0.01, **A**). RR also significantly decreased NO products in irradiated *ρ*^+^ donor cells (*P*<0.05, **B**). Data are pooled from at least three independent experiments and the results represent mean±s.d.

The activation of mitochondrial nitric oxide synthase (mtNOS) is known to be Ca^2+^ dependent. Consequently, RR and calmidazolium were used to further corroborate the role of calcium signalling in bystander effects. As shown in Figure 3A, pretreatment of cells with RR or calmidazolium significantly reduced the *γ*-H2AX induction of *ρ*^+^ ICCM (*P*<0.01; Figure 3A). Moreover, treatment with RR also significantly decreased NO products in *ρ*^+^ donor cells (*P*<0.05; Figure 3B). These data suggest that radiation-induced calcium flux is necessary for the induction of the bystander *γ*-H2AX, probably through the activation of mtNOS on the inner membranes of mitochondria.

### Radiation stimulates cellular O_2_^•−^ production in irradiated *ρ*^+^ A_L_ cells, but not in *ρ*^0^ A_L_ cells

**Figure 4:**
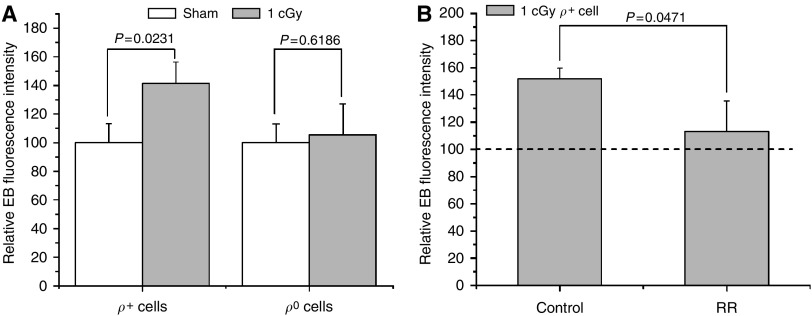
Effects of irradiation on the EB fluorescence intensity. The relative EB fluorescence intensities in 1 cGy-irradiated *ρ*^+^ A_L_ cells were significantly increase in 10 min after irradiation (*P*<0.05), whereas there was no significant increase in 1 cGy-irradiated *ρ*^0^ A_L_ cells (*P*>0.05, *n*=3, **A**). Pretreatment with 10 *μ*m RR reduced the relative EB to background levels in 1 cGy-irradiated *ρ*^+^ A_L_ cells in 10 min after irradiation (*P*<0.05, **B**). Data are pooled from at least three independent experiments, and the results are represented as mean±s.d.

To monitor the levels of O_2_^•−^ in *ρ*^+^ or *ρ*^0^ A_L_ cells after irradiation, the fluorescent probe, dihydroethidine, was employed. Figure 4A showed that the relative fluorescence intensity of ethidium bromide (EB), the fluorescent product of dihydroethidine when reacted with O_2_^•−^, was significantly increased in 1 cGy-irradiated *ρ*^+^ A_L_ cells comparing with the sham-irradiated control (*P*<0.05) and the increased EB fluorescence intensities were derived from superoxide anion (Supplementary Figure 1). In contrast, there was no significant increase EB 1 cGy-irradiated *ρ*^0^ A_L_ cells (*P*>0.05). Moreover, treatment with RR also significantly decreased O_2_^•−^ products in *ρ*^+^ donor cells (*P*<0.05; Figure 4B). This result provides further evidence that radiation-induced ROSs are derived from mitochondria and mitochondrial calcium uptake upregulate ROS products.

## DISCUSSION

Mitochondria, which are ubiquitous in the cytoplasm of a cell, are important targets of radiation-induced cellular responses including apoptosis and oxyradical production (Belka *et al*, 2000; Leach *et al*, 2001). The prominence of mitochondrial function in radiation response has become more significant after bystander effects were observed in experiments of cytoplasmic irradiation (Wu *et al*, 1999; Shao *et al*, 2004). These studies suggest that direct nuclear damage is not required for switching on cell-signalling mechanisms (Morgan and Sowa, 2006) and damage to cytoplasm can also generate damage signals. Thus, the critical question is how the damage or damage signals are generated and whether it is related to mitochondrial damage as a result of cytoplasmic irradiation. In the present study, using mtDNA-depleted and normal cells and the induction of *γ*-H2AX foci as a biological end point (Burdak-Rothkamm *et al*, 2007) in nonirradiated AG1522 cells, we investigated the role of mitochondria in the early process of RIBE. The results indicate that mtDNA-depleted cells or normal A_L_ cells treated with mitochondrial respiratory chain function inhibitors have an attenuated *γ*-H2AX induction (Figure 1A and B), which clearly suggest that mitochondria play a crucial role in the early processes of RIBE. Moreover, our results using A_L_ cells as donor cells and human fibroblasts as receptor cells, clearly illustrate that in the early process, RIBE is not cell-type dependent and the signal can be transferred across species. Furthermore, the relatively low *γ*-H2AX induction by irradiated medium from *ρ*^0^ cells suggests that other signal production pathways are likely to be involved in the early processes of RIBE.

The role of soluble transmissible factor(s), such as ROSs, RNSs, and cytokines generated by irradiated cells that in turn induce toxic effects in nonirradiated cells, has been demonstrated by many medium transfer experiments (Mothersill and Seymour, 1998; Lyng *et al*, 2000). Leach *et al* (2002) observed that activation of cNOS activity was an early signal event after irradiation. Recent studies have demonstrated the important role of constitutive NO in mediating the early bystander responses induced by low-dose irradiation (Han *et al*, 2007a). In the present study using inhibitors of NOS, including specific inhibitor of cNOS, we found that, consistent with our previous observations with AG1522 cells, the *γ*-H2AX induction of *ρ*^+^ ICCM was significantly decreased (Figure 2A) and ionising radiation stimulated cellular NO production in irradiated *ρ*^+^ A_L_ cells, but not in *ρ*^0^ A_L_ cells (Figure 2B). These results suggest that activity of NOS in *ρ*^+^ A_L_ cells, especially cNOS, is involved in the early processes of RIBEs and mitochondria might be a main source in the generation of NO.

Using a variety of approaches including immunohistochemistry, western blotting, fluorescent staining, NOS^−/−^ mice, and microsensors, mtNOS has been shown to be distributed in diverse organs of many species (Bates *et al*, 1995; Kanai *et al*, 2001, 2004; Kato and Giulivi, 2006; Ghafourifar and Sen, 2007). Changes in mtNOS expression and activity in many pathophysiological situations may imply its significant involvement in various NO-related biological phenomena. In contrast, there have been reports that raised doubts on the existence of mtNOS based on methodological issues, and speculated that eNOS, which is attached to the outer mitochondrial membrane, mediates the release of NO (Lacza *et al*, 2006). However, this hypothesis is very difficult to explain, as NO production can be significantly decreased by preventing mitochondrial Ca^2+^ uptake. Recently, Ghafourifar and his colleagues have reported that hypoxia/reoxygenation and tamoxifen can elevate [Ca^2+^]_m_ by increasing mitochondrial calcium uptake and the release of [Ca^2+^]_m_ from the granules in mitochondria, and stimulate mtNOS to generate NO (Nazarewicz *et al*, 2007; Zenebe *et al*, 2007). By inhibiting mitochondrial calcium uptake with RR, we found that the relative fluorescence intensity of NO significantly decreased in post-irradiated *ρ*^+^ A_L_ cells (Figure 3B). Moreover, treatment with RR resulted in a significant decreased in *γ*-H2AX induction of *ρ*^+^ ICCM (Figure 3A). These results suggest that mitochondrial calcium uptake and the activation of mtNOS might be an important event in producing bystander factor(s).

It has been reported that ROS scavenger, such as dimethyl sulphoxide, superoxide dismutase, and catalase, can effectively suppress RIBE (Narayanan *et al*, 1997; Hu *et al*, 2005). In the present study, O_2_^•−^ levels were significantly increased in *ρ*^+^ A_L_ cells after irradiation, but not in *ρ*^0^ A_L_ cells (Figure 4A), and RR decreased the O_2_^•−^ levels in irradiated *ρ*^+^ A_L_ cells (Figure 4B). The generation of O_2_^•−^ might also be related, at least partially, to mitochondria in the irradiated normal cells. Although inhibitors of mitochondrial respiratory chain, such as rotenone, antimycin A, and oligomycin, may cause ROS production, these inhibitors also affect the activity of mtNOS and decrease radiation-induced NO products. Dedkova *et al* (2004) reported that inhibition of mitochondrial respiratory chain decreased mitochondrial NO production. Using dihydrodichlorofluorescein to determine the ROS/RNS production, Leach *et al* (2001) observed that rotenone decreased radiation-induced ROS/RNS production. These studies suggested that the activity of the respiratory chain might play an important role in the regulation of mtNOS (Dedkova *et al*, 2004) and essential components of mitochondrial respiratory chain might be cofactors, which are requires by activation of mtNOS (Bates *et al*, 1996). Moreover, inhibitors of mitochondrial respiratory chain may collapse the mitochondrial membrane potential, which will decrease the mitochondrial calcium uptake and affect generation of NO by mtNOS. The relationship between radiation-induced ROS and RNS is complex, both of them are important to initiate bystander effects. Inhibitions of mitochondrial respiratory chain increase ROS, but decrease NO, and result in attenuated bystander *γ*-H2AX (Figure 1B).

In summary, based on our data and those of others, a working model on how mitochondrial function contributes to RIBE can be postulated. Exposure of cells to ionising irradiation stimulates a reversible mitochondrial permeability transition (Leach *et al*, 2001), which occurs during activation of permeability pathways in the inner mitochondrial membrane and stimulates mitochondrial Ca^2+^ uptake (Kanai *et al*, 2004). The increased [Ca^2+^]_m_ will activate mtNOS to produce NO. The elevated NO level will inhibit cytochrome oxidase (complex IV) in the respiratory chain and increases O_2_^•−^ formation by coenzyme Q (Beltran *et al*, 2002). The increased ROS will in turn caused a biphasic increase in [Ca^2+^]_m_ level that will continue to stimulate production of NO and O_2_^•−^, both of which, in part, will react and form peroxynitrite ion (ONOO^−^). The ONOO^−^ can act with protein and DNA that causes continued cellular responses, including later process of bystander. This ring-like generation of NO in mitochondria by ionising radiation will penetrate cellular membranes as an intercellular signalling molecule, and, finally, results in damages in nonirradiated bystander cells in early process of RIBE.
