# Supplementary material for: Mitochondria-dependent signalling pathway are involved in the early process of radiation-induced bystander effects
Source: Br J Cancer. 2008 May 13;98(11):1839–44. doi: 10.1038/sj.bjc.6604358 (PMC2410123; doi:10.1038/sj.bjc.6604358)
Supplement: Supplementary Figure 1 [file 6604358x1.doc]

**Supplemental data**

**Superoxide dismutase decreases the relative EB fluorescence intensities and NaN3 has no effects on the relative DAF-triazole fluorescence intensities**

As Dihydroethidine can be also oxidized by cytochrome *c* and ONOO^-^, and DAF-DA can be nitrosated by N_2_O_3_, yielding superoxide-independent or NO-independent fluorescence, we pre-treated the cultures with superoxide dismutase, with specific activity in the reduction of O_2_^.-^ to hydrogen peroxide (SOD, 100μg/ml, Sigma, USA) and NaN_3_ (1mM, scavenger of N_2_O_3_), respectively. After treatment with SOD, the relative EB fluorescence intensities in ρ^+^ A_L_ cells irradiated with a 1cGy α-particles significantly decreased relative to controls (1.25±0.12 folds for 1cGy-irradiated group and 1±0.10 folds for SOD-treated group, p<0.05) (figure 1a), providing evidence that the increased EB fluorescence intensities were derived from O_2_^.-^. Moreover, in ρ^+^ A_L_ cells treated with NaN_3_, there were no significant difference in the relative DAF-triazole fluorescence intensities detected (1.16±0.03 folds for 1cGy-irradiated group, and 1.13±0.07 folds for NaN_3_-treated group, p>0.05) (figure 1b). These data suggest that the increased fluorescence are caused by NO and these results further confirm the important role of mitochondria in the induction of NO and O_2_^.-^ during the early phase of the bystander response.


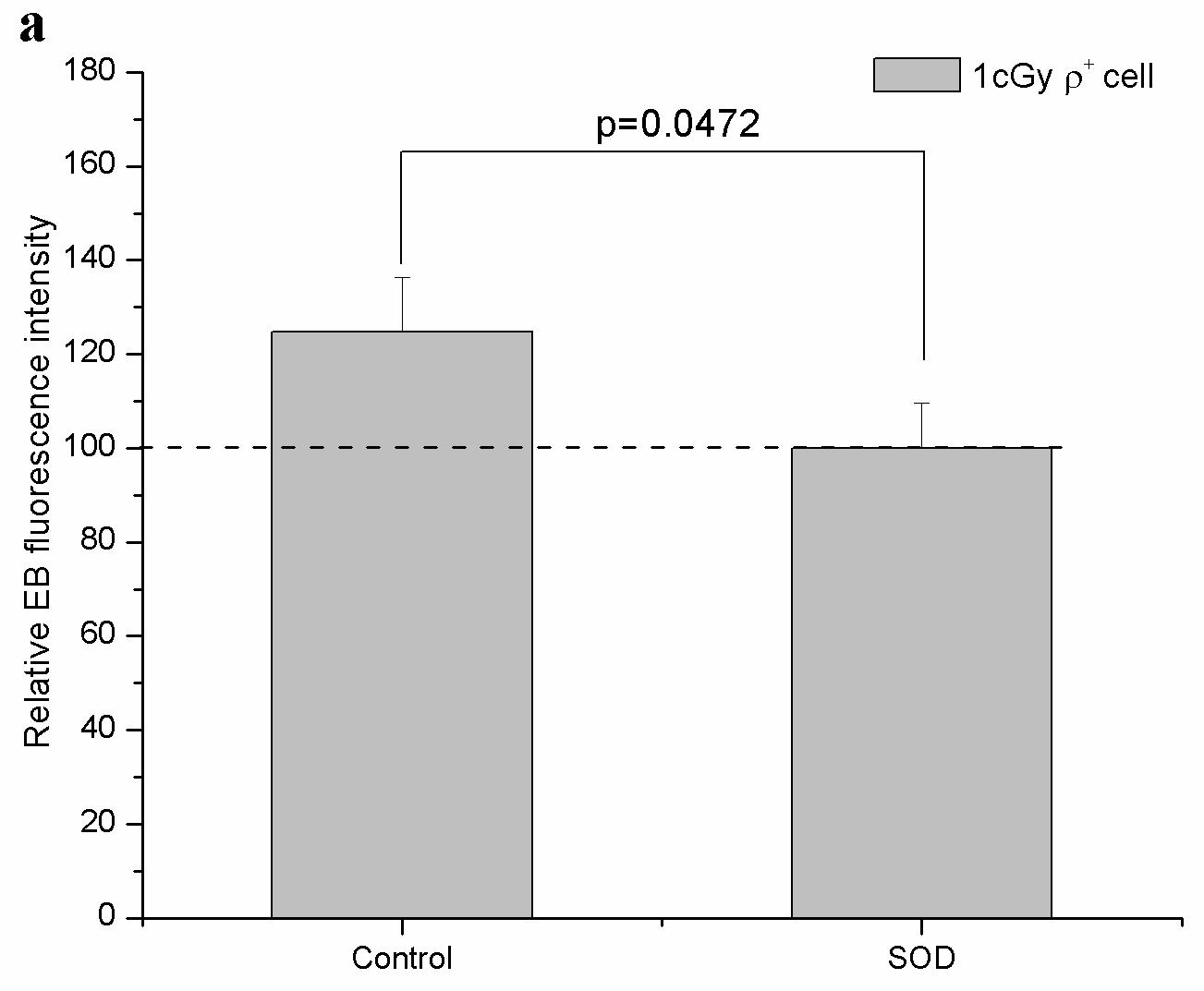


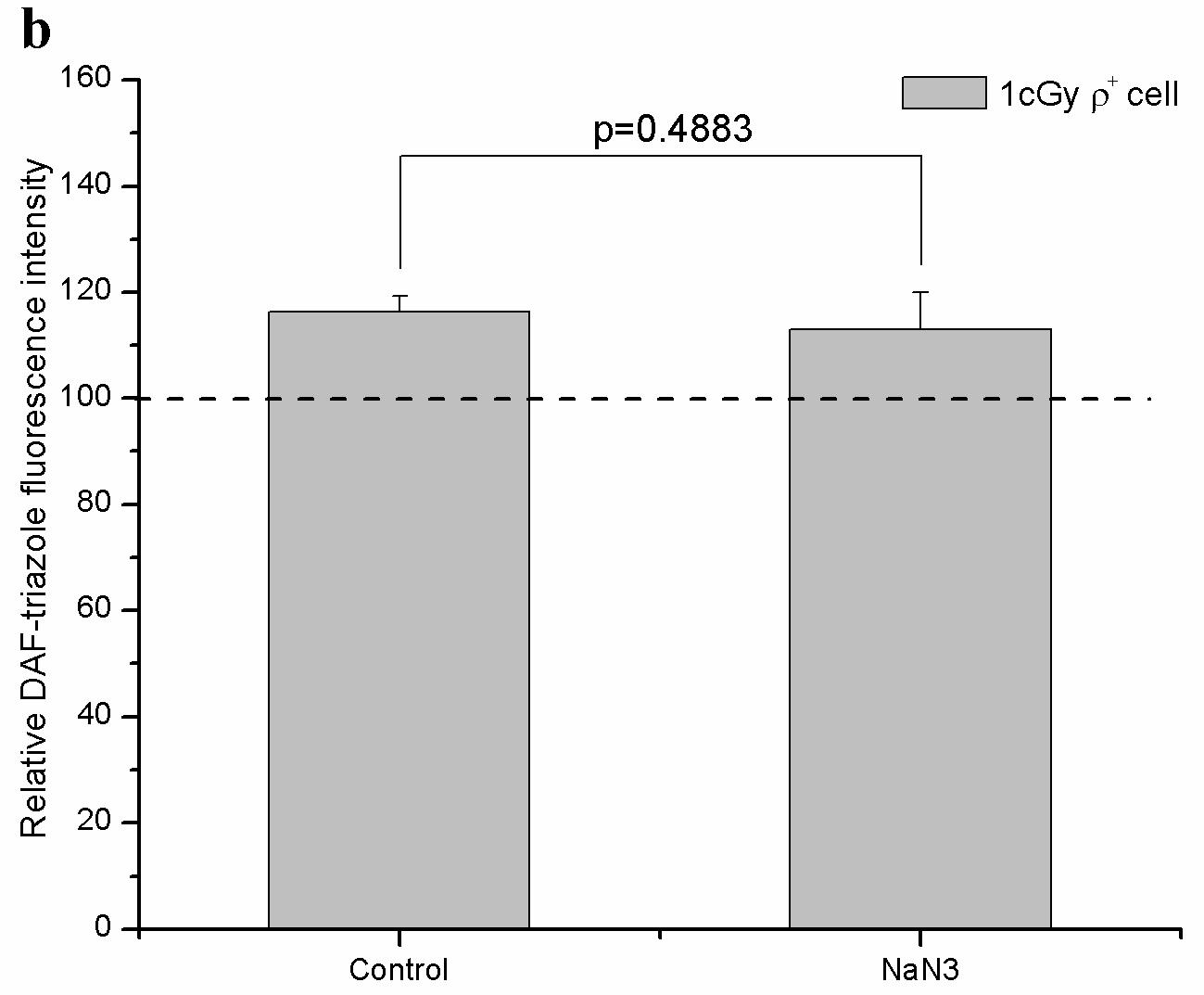


Figure 1 Effect of SOD/NaN_3_ on the EB/DAF-triazole fluorescence intensity in 1cGy-irradiated ρ^+^ A_L_ cells. SOD treatment significantly decreased the EB fluorescence intensities (Fig. 1a, p<0.05) and NaN_3_ couldn’t significantly change the DAF-triazole fluorescence intensities (Fig. 1b, p>0.05), indicating that the relative fluorescence intensities caused by O_2_^.-^ and NO respectively. Data are pooled from at least three independent experiments, and the results are represented as mean±s.d.
